# Targeted therapy and biomarker-guided applications of ecofriendly silver nanoparticles in precision oncology

**DOI:** 10.3389/jpps.2025.15403

**Published:** 2026-01-13

**Authors:** Haider Hamzah

**Affiliations:** Department of Biology, University of Sulaimani, Sulaymaniyah, Kurdistan, Iraq

**Keywords:** cancer therapy, precision oncology, silver nanoparticles, targeted drug delivery, theranostics

## Abstract

Eco-friendly silver nanoparticles (eco-AgNPs) represent a promising convergence of green nanotechnology and precision medicine for cancer treatment. This minireview examines the therapeutic potential of silver nanoparticles (AgNPs) synthesized through eco-friendly methods using plant extracts and microorganisms. These eco-friendly AgNPs demonstrate enhanced biocompatibility and selective cytotoxicity against malignant cells. These nanoparticles target cancer through multiple mechanisms including reactive oxygen species generation, apoptosis induction, and cell cycle disruption. Selectivity is achieved through surface functionalization with targeting moieties such as antibodies and aptamers that recognize overexpressed tumor receptors. The integration of biomarker-guided design enables tumor-specific delivery by exploiting unique metabolic signatures and cellular markers characteristic of different cancer types. Furthermore, AgNP-based theranostic platforms offer simultaneous diagnostic imaging and therapeutic intervention, providing real-time assessment of treatment response and enabling personalized dosing strategies. However, clinical translation faces significant challenges including potential long-term toxicity, standardization of synthesis protocols, and regulatory approval pathways. Successful clinical implementation will require interdisciplinary collaboration to optimize nanoparticle design, establish safety profiles, and develop combination therapies that maximize therapeutic benefits while minimizing side effects. Eco-AgNPs thus offer a transformative approach to cancer treatment that combines environmental sustainability with precision targeting capabilities.

## Introduction

Precision oncology has transformed cancer treatment from generalized approaches to personalized strategies guided by tumor genetics and molecular biomarkers [1]. This paradigm maximizes therapeutic efficacy while minimizing adverse effects through targeted interventions. Nanotechnology has further advanced precision oncology by enabling novel drug delivery systems with enhanced bioavailability and tumor-specific targeting [2]. Among nanomaterials, eco-friendly silver nanoparticles (eco-AgNPs) are particularly promising, owing to their unique physicochemical properties and inherent antiproliferative activity [3–6]. While chemically synthesized AgNPs offer precise control over size and morphology, their reliance on toxic reducing agents (e.g., sodium borohydride) raises concerns about residual toxicity and environmental impact [7, 8]. In contrast, eco-AgNPs employ natural capping agents from plant or microbial extracts, such as *Fusarium oxysporum* [9] and *Aeromonas caviae* [10], enhancing biocompatibility and reducing off-target effects. This green synthesis approach aligns with sustainable chemistry principles, albeit with greater batch-to-batch variability [11]. This green synthesis approach aligns with sustainable chemistry principles while providing a versatile platform for cancer theranostics that balances efficacy with reduced toxicity.

Eco-AgNPs demonstrate multifaceted anticancer mechanisms distinct from conventional chemotherapeutics. Through sustained Ag^+^ ion release, mitochondrial disruption, DNA/protein interactions, and ROS generation, they induce selective apoptosis in cancer cells [12]. This multimodal action may overcome drug resistance associated with single-target therapies. Their biogenic capping (e.g., peptides, polysaccharides) not only improves stability and biocompatibility [13, 14] but also enables functionalization with targeting ligands (antibodies, aptamers) for biomarker-specific delivery [15]. While protein corona formation in biological fluids presents challenges, it also offers opportunities to engineer cellular interactions for enhanced therapeutic outcomes [16].

This mini-review critically evaluates eco-AgNPs as precision oncology tools, examining their anticancer mechanisms, targeted functionalization strategies, and biomarker-driven applications. We discuss key challenges in therapeutic translation, emphasizing their potential to address tumor heterogeneity while minimizing systemic toxicity. By connecting mechanistic understanding with clinical realities, this analysis highlights eco-AgNPs’ emerging role in personalized cancer therapy.

## Mechanisms of action of eco-AgNPs in cancer therapy

Eco-friendly silver nanoparticles (eco-AgNPs) exert their anticancer effects through a multifactorial approach, engaging multiple cellular pathways simultaneously. This multi-pronged attack represents a key advantage, as cells with deficient DNA repair mechanisms show increased sensitivity to AgNPs compared to cells with intact repair systems [17]. Nanomaterial, such as eco-AgNPs, enter cancer cells primarily through endocytosis, bypassing drug efflux pumps responsible for multidrug resistance [18]. Their passive accumulation in solid tumors is facilitated by the enhanced permeability and retention (EPR) effect, a foundational principle of nanomedicine that provides an initial layer of tumor specificity. Once internalized, eco-AgNPs are trafficked to acidic organelles like lysosomes, where the low pH environment promotes their dissolution and the sustained release of highly reactive silver ions (Ag^+^) [19]. These ions, along with the nanoparticles themselves, then initiate a cascade of cytotoxic events.

The primary mechanism of eco-AgNPs across different cancer types involves the robust generation of reactive oxygen species (ROS), including superoxide anions and hydroxyl radicals, leading to oxidative damage of lipids, proteins, and DNA [12, 20]. Notably, eco-AgNPs demonstrate potent cytotoxic efficacy, with reported IC_50_ values as low as 5.44 μg/mL against MCF-7 breast cancer cells [21]. This inherent anticancer activity is significant, as it can be achieved without conjugation to conventional chemotherapeutics. For context, some chemically synthesized AgNP-drug delivery systems, such as those conjugated with 5-fluorouracil (5FU), have reported higher IC_50_ values (e.g., 23.006 μg/mL in the same cell line), which underscores the powerful standalone potential of certain ecofriendly nanoparticle formulations [22]. This counter-intuitive finding suggests that covalent conjugation of chemotherapeutics might occasionally alter the nanoparticle’s physicochemical surface properties-specifically the bio-corona-potentially hindering cellular uptake or modifying the release kinetics of silver ions compared to the pristine, biologically capped eco-AgNP. This therapeutic efficacy of eco-AgNPs is attributed to their naturally derived bio-corona, which not only enhances biocompatibility and reduces off-target toxicity but also facilitates more efficient cellular internalization and sustained intracellular silver ion (Ag^+^) release. The resulting enhanced ROS generation is particularly relevant to precision oncology, as cancer cells often have compromised antioxidant systems and lower total antioxidant capacity [23], making them selectively more susceptible to ROS-inducing agents compared to healthy cells [21, 24]. This vulnerability arises because cancer cells typically operate under higher basal oxidative stress due to accelerated metabolism (the Warburg effect), leaving them with a depleted antioxidant reserve compared to normal tissues. A key consequence of this oxidative stress is the specific targeting of mitochondria. Eco-AgNPs disrupt the mitochondrial membrane potential and uncouple the electron transport chain, triggering the release of pro-apoptotic factors such as cytochrome c into the cytoplasm [12]. This subsequently activates initiator and executioner caspases (e.g., caspase-3, -7, -8, -9), initiating the intrinsic apoptotic pathway and leading to programmed cell death [25]. Concurrently, eco-AgNPs induce significant genotoxicity by causing DNA double-strand breaks and interfering with replication and repair mechanisms. This activates cell cycle checkpoints and leads to robust G0/G1 or G2/M arrest, thereby halting cancer cell proliferation [21].

**FIGURE 1:**
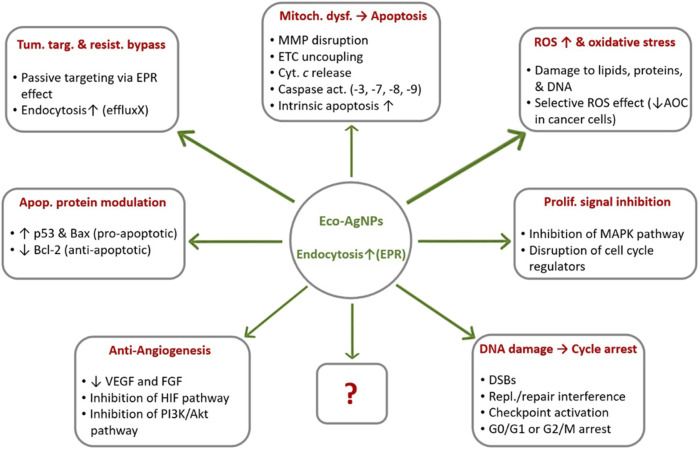
Multimodal anticancer mechanisms of ecofriendly silver nanoparticles (eco-AgNPs). Note: The question mark (?) in the figure indicates other unknown mechanisms that may be revealed by future studies owing to the multi-targeted actions of eco-AgNPs. Abbreviations: AOC, Antioxidant Capacity; DSB, DNA Double-Strand Breaks; EPR, Enhanced Permeability and Retention; ETC, Electron Transport Chain; MMP, Mitochondrial Membrane Potential; ROS, Reactive Oxygen Species. ↑, upregulation/enhancement; ↓, inhibition/downregulation (Created by the author and adapted from [12, 21, 25, 27]).

Beyond these ROS-mediated effects, eco-AgNPs exert their anticancer effects through multiple synergistic mechanisms. Their interactions with critical signaling proteins enable modulation of apoptotic pathways, including upregulation of p53 and Bax alongside downregulation of Bcl-2 [25, 26]. Furthermore, they inhibit tumor proliferation by disrupting cell cycle progression and interfering with MAPK signaling [2]. Importantly, eco-AgNPs also target tumor vascularization by suppressing VEGF and FGF expression while impairing HIF and PI3K/Akt pathways [27, 28]. This multifaceted action against apoptosis, proliferation, and angiogenesis underscores their potential as precision oncology therapeutics. The multifactorial anticancer mechanisms of eco-AgNPs are summarized in Figure 1. These nanoparticles induce cancer cell death through both direct cytotoxic effects, such as ROS generation, mitochondrial dysfunction, including mitochondrial membrane potential (MMP) loss and electron transport chain (ETC) disruption, and DNA double-strand breaks (DSBs), and regulatory effects, including caspase activation, p53/Bax upregulation, Bcl-2 downregulation, and VEGF pathway suppression. Their selective toxicity toward cancer cells is attributed to the lower antioxidant capacity (AOC) of malignant cells and the enhanced permeability and retention effect (EPR) effect.

## Targeted nanotherapeutics: functionalization strategies

The transition from passive to active targeting represents a paradigm shift in nanomedicine, enabling precise delivery of therapeutic agents to specific cellular targets while minimizing off-target effects. For eco-AgNPs in precision oncology, surface functionalization strategies have emerged as a critical approach to enhance therapeutic specificity and overcome the limitations of conventional passive targeting mechanisms [29]. The inherent advantage of eco-AgNPs lies in their naturally occurring bio-corona, which provides multiple functional groups and binding sites that can be exploited for targeted modification without compromising nanoparticle stability or biocompatibility [11, 30]. While passive targeting through the EPR effect provides initial tumor accumulation, active targeting strategies offer superior precision by exploiting the molecular signatures of cancer cells [31]. Passive targeting relies on the anatomical and physiological differences between tumor and normal tissues, including enhanced vascular permeability, defective lymphatic drainage, and prolonged retention times [32]. However, the heterogeneous nature of tumor vasculature and the variability in EPR effects across different cancer types limit the clinical efficacy of passive targeting alone [33]. Active targeting addresses these limitations by incorporating specific ligands that recognize and bind to biomarkers overexpressed on cancer cell surfaces, thereby facilitating receptor-mediated endocytosis and enhancing intracellular drug delivery [34].

The selection of appropriate targeting ligands is crucial for achieving selective cancer cell recognition and uptake. Antibodies represent the most extensively studied targeting ligands, offering high specificity and affinity for their cognate antigens [35]. Although chemical synthesis allows easier surface modification due to predictable ligand conjugation (e.g., via thiol chemistry), eco-AgNPs’ inherent biomolecular corona (e.g., proteins, polysaccharides) can serve as a natural platform for functionalization, reducing the need for additional coating steps and potentially improving *in vivo* stability [11]. Monoclonal antibodies targeting overexpressed receptors such as human epidermal growth factor receptor 2 (HER2) in breast cancer [36], epidermal growth factor receptor (EGFR) in lung and oral cancers [37, 38] and CD20 in lymphomas [39] have been successfully conjugated to both chemically synthesized and eco-AgNPs, demonstrating enhanced therapeutic efficacy and reduced systemic toxicity [2]. However, the large molecular size of antibodies may limit tissue penetration and increase immunogenicity, necessitating the development of smaller alternatives [40]. On the other hand, peptide-based targeting ligands offer several advantages over antibodies, including smaller size, lower immunogenicity, enhanced tissue penetration, and cost-effective synthesis [41]. Furthermore, tumor-homing peptides such as RGD (Arg-Gly-Asp) sequences that target αvβ3 integrins, and tumor-penetrating peptides like iRGD (internalizing RGD) that facilitate deep tissue penetration, have been successfully employed to functionalize AgNPs for targeted cancer therapy [42–44]. These peptides can be synthesized with specific amino acid sequences that confer selectivity for particular cancer types or stages, enabling personalized therapeutic approaches [45]. Interestingly, aptamers, short single-stranded DNA or RNA molecules that bind to specific target proteins with high affinity, represent another promising class of targeting ligands. Their unique advantages include small size (typically 8–15 kDa), chemical stability, lack of immunogenicity, and the ability to be chemically modified for enhanced functionality [46]. Aptamers targeting cancer-associated proteins such as nucleolin, mucin 1, and prostate-specific membrane antigen have been conjugated to various types of nanoparticles, demonstrating selective cancer cell binding and internalization [47], suggesting potential applications for eco-AgNP conjugation.

The effective bioconjugation of targeting moieties to eco-AgNPs demands sophisticated surface modification approaches that simultaneously maintain nanoparticle structural integrity and preserve the biological activity of conjugated ligands. Covalent conjugation strategies, including carbodiimide chemistry, maleimide-thiol coupling, and click chemistry, provide stable linkages between targeting ligands and nanoparticle surfaces [48]. The choice of conjugation method depends on the available functional groups on both the nanoparticle surface and the targeting ligand [15]. Eco-AgNPs possess an inherent bio-corona enriched with multiple functional groups, notably amine, carboxyl, and hydroxyl moieties, which provide accessible conjugation sites for ligand immobilization [30]. Spacer molecules and linkers play a crucial role in maintaining the biological activity of conjugated ligands by preventing steric hindrance and providing optimal orientation for target recognition [49]. Polyethylene glycol (PEG) linkers are commonly employed to enhance the flexibility and accessibility of targeting ligands while also providing stealth properties that reduce protein adsorption and extend circulation time [50]. The length and composition of these linkers can be optimized to achieve the desired balance between targeting efficiency and nanoparticle stability [51]. Advanced functionalization strategies incorporate stimuli-responsive elements that enable controlled drug release in response to specific tumor microenvironmental conditions. pH-responsive systems exploit the acidic environment of tumor tissues (pH 6.5–7.0) and endosomal compartments (pH 5.0–6.0) to trigger targeted drug release [52, 53]. pH-sensitive linkages, such as hydrazone bonds and acid-labile acetals, can be incorporated into the targeting ligand conjugation to achieve selective drug release at the tumor site [54]. Furthermore, enzyme-responsive targeting systems utilize the overexpression of specific enzymes in tumor tissues, such as matrix metalloproteinases (MMPs) and cathepsins, to trigger drug release [55]. Peptide substrates that are specifically cleaved by these enzymes can be incorporated as linkers between targeting ligands and drug payloads, enabling precise spatial and temporal control of therapeutic agent release [56].

Despite the promising potential of targeted eco-AgNP therapeutics, several challenges must be addressed to optimize their clinical translation, including tumor heterogeneity, optimization of ligand density, and quality control of functionalized nanoparticles [57–59]. Multi-ligand functionalization approaches and advanced characterization techniques are essential for overcoming these limitations and ensuring therapeutic efficacy. The development of targeted AgNP therapeutics represents a convergence of nanotechnology, molecular biology, and precision medicine, offering unprecedented opportunities for personalized cancer therapy that will undoubtedly play an increasingly important role in next-generation precision oncology therapeutics.

## Biomarker-driven applications in precision oncology

In precision oncology, biomarkers function as quantifiable biological indicators that inform cancer diagnosis, prognosis, and treatment selection, facilitating the implementation of personalized therapeutic strategies [60]. Eco-AgNPs, with their highly tunable surface properties, are uniquely positioned to be engineered to utilize these biomarkers, transforming them from general cytotoxic agents into highly selective, targeted delivery platforms [61, 62]. Strategic functionalization enables eco-AgNPs to bind specifically to receptors or proteins overexpressed on cancer cell surfaces while exhibiting minimal affinity for healthy cells. This selective targeting forms the cornerstone of biomarker-driven precision oncology. One predominant approach in biomarker-guided therapy involves targeting overexpressed growth factor receptors on cancer cell surfaces. Receptors such as the epidermal growth factor receptor (EGFR) and human epidermal growth factor receptor 2 (HER2) are well-established drivers in lung, breast, and gastric cancers [63, 64]. In preclinical *in vitro* studies, nanoparticles have been successfully functionalized with ligands like antibodies and affibodies to achieve active tumor targeting. Specifically, EGF-labeled liposomes have been used to direct eco-AgNPs to EGFR-overexpressing cells [63], while various nanotherapeutics have been conjugated with antibodies like Trastuzumab to target HER2-positive breast cancers [36, 64]. To illustrate these applications, Table 1 summarizes key biomarkers and their associated targeting strategies; while many of these foundational approaches were established using various nanoparticle systems, they provide a validated blueprint for engineering the next-generation of eco-AgNP-based therapeutics. Among the most extensively studied biomarkers are the growth factor receptors EGFR and HER2, which are frequently overexpressed across aggressive cancers, including pancreatic, breast, and lung malignancies. Eco-AgNPs functionalized with anti-EGFR antibodies or HER2-targeting affibodies have demonstrated promising therapeutic effects in preclinical studies [66]. In addition to these broadly relevant receptors, pancreatic adenocarcinoma (PDAC) presents unique targeting opportunities through several overexpressed biomarkers. Mesothelin, a membrane protein crucial for cell survival, migration, and invasion, has become a focus for both diagnostic imaging and therapeutic intervention, with nanoparticle imaging probes and mesothelin antibody-conjugated liposomes under active development [65]. The urokinase plasminogen activator receptor (uPAR) represents another attractive target, as it is highly expressed in both pancreatic cancer cells and their surrounding stromal cells, making it particularly valuable for improving intratumoral drug delivery and ensuring therapeutic agents reach difficult-to-penetrate tumor regions [69]. The urokinase plasminogen activator receptor (uPAR) is another compelling biomarker, as it is highly expressed in both pancreatic cancer cells and their surrounding stromal cells. Targeting uPAR is particularly valuable for improving the delivery of therapeutic agents into dense, hard-to-penetrate tumors. A recent preclinical study demonstrated that an antibody-drug conjugate (ADC) targeting uPAR successfully suppressed tumor growth in pancreatic cancer models [69]. While this study employed an ADC rather than a nanoparticle system, the validation of this target provides a compelling blueprint for future eco-AgNP conjugation strategies. [69]. While this study did not use nanoparticles, it confirms uPAR as a high-potential target for future AgNP-based delivery strategies. Additional emerging biomarkers in pancreatic cancer include Plectin-1, Mucin-1, and ZIP4, which are being explored for targeted imaging and therapeutic interventions, reflecting the growing sophistication of eco-AgNP applications in highly specific cancer types [70]. Moreover, beyond traditional tumor markers, CD44 has gained attention as a common cancer stem cell marker that can be effectively targeted by AgNPs [68]. This targeting strategy impacts the expression of genes related to cancer stem cells and potentially offers a pathway to overcome drug resistance [22, 71]. The ability to target cancer stem cells represents a particularly promising avenue, as these cells are often responsible for treatment resistance and tumor recurrence [72].

**TABLE 1:** Key cancer biomarkers and their targeted applications with nanoparticles/eco-AgNPs in precision oncology.

Biomarker	Cancer type	Role in pathogenesis	NPs/AgNP^a^ targeting strategy	Application type	Preclinical efficacy/outcome	References
Mesothelin	Pancreatic, ovarian, mesothelioma	Cell survival, migration, invasion, progression	Antibody conjugation (e.g., mesothelin-targeted liposomes)	Targeted therapy, diagnostic imaging, theranostics	Simultaneous detection & therapy; improved drug delivery	[65]
EGFR	Pancreatic, lung, breast	Cell growth, proliferation, drug resistance	Antibody conjugation (e.g., Anti-EGFR)	Targeted therapy	Inhibited growth, apoptosis, increased radiation sensitivity	[37]
HER2	Breast, gastric	Cell growth, proliferation, drug resistance	Affibody conjugation (e.g., HER2-affibody)	Targeted therapy	High binding, cytotoxicity, tumor growth inhibition	[22, 66]
Lactate^b^	Various solid tumors	Altered glucose metabolism, energy production	Glucose-functionalized AgNPs (G-AgNPs)	Targeted therapy, drug delivery	Enhanced cytotoxicity, DNA damage	[67]
CD44	Lung, cancer stem cells	Stem cell marker, adhesion, migration	Ligand-mediated targeting (e.g., anti-CD44)	Targeted therapy	Mitochondrial damage, apoptosis, and autophagy	[68]
Plectin-1	Pancreatic ductal adenocarcinoma	PDAC-specific overexpression	Nanoparticle imaging probes	Diagnostic imaging, targeted therapy	Potential for early diagnosis and intervention	[65]

^a^ The references found in this table deal with numerous nanoparticles, some of which are eco-AgNPs.

^b^ Warburg effect.

Beyond surface proteins, metabolic biomarkers also offer compelling avenues for targeted intervention. The “Warburg effect,” characterized by elevated glucose metabolism and lactate production in cancer cells, can be exploited for targeted delivery using glucose-functionalized AgNPs [67]. This targeting strategy exploits the overexpression of specific receptors on cancer cell surfaces, such as folate receptors, through functionalization with targeting ligands including antibodies, peptides, and aptamers. This approach enables selective binding and preferential accumulation of AgNPs in tumor cells that overexpress these target receptors, while minimizing uptake in healthy cells with lower receptor expression levels [66]. Building upon these targeting strategies, theranostics represents a powerful extension that integrates diagnostic imaging with therapeutic delivery within a single nanoparticle platform. This approach enables real-time, non-invasive monitoring of drug delivery, biodistribution, and treatment response, representing a significant advancement over traditional oncology where treatment efficacy is assessed retrospectively [73]. Eco-AgNPs can serve as contrast agents for enhanced tumor imaging, guiding surgeons for more accurate tumor removal or improving contrast in modalities like MRI [2]. Specific applications include mesothelin antibody-conjugated liposomes loaded with iron oxides and doxorubicin for simultaneous detection and therapy of pancreatic cancer [65]. The advantages of eco-AgNPs for theranostics include their biocompatibility, stability, and ability to facilitate proteogenomic imaging for tracking cellular activity [74]. This integrated approach provides real-time insights into drug delivery and therapeutic effects, facilitating dynamic treatment adjustments and ultimately leading to improved patient outcomes with reduced side effects [2, 75].

Ultimately, the detection of high levels of specific biomarkers in a patient’s tumor is crucial for biomarker-guided patient stratification, allowing clinicians to select the most appropriate targeted therapy and move towards truly personalized medicine. This approach promises to improve treatment efficacy and reduce adverse effects by ensuring therapies are delivered to patients most likely to benefit. However, it is important to acknowledge that some biomarkers may not be entirely cancer-specific, and tumor heterogeneity remains a persistent challenge. This complexity means that targeting a single biomarker might not always be sufficient for complete tumor eradication, potentially necessitating multi-biomarker targeting or combination therapies to overcome the adaptive nature of cancer [76]. While the strategies mentioned above have been validated, the principles demonstrated in other advanced nanomedicine systems highlight promising future directions for eco-AgNP-based therapies. For instance, the acidic tumor microenvironment could be exploited by developing pH-responsive AgNPs designed for acid-triggered release, a mechanism that has been successfully demonstrated *in vitro* and *in vivo* for other nanocarriers [53]. Similarly, in cancers with genetic vulnerabilities like BRCA1/2 mutations, combining AgNPs with PARP inhibitors could create a synthetic lethality approach, a strategy that is being explored with other nanoparticle systems [77]. Furthermore, the theranostic potential of AgNPs could be expanded by developing platforms with integrated imaging probes for real-time monitoring of therapy, an approach that has shown success for HER2-positive cancers using different types of nanoparticles [78]. Validating these advanced strategies specifically for AgNPs is a critical next step to broaden their application in precision oncology.

## Current challenges and future perspectives

**FIGURE 2:**
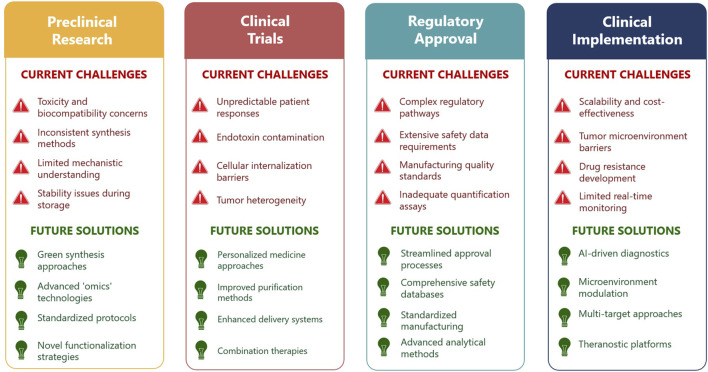
Current challenges and future perspectives in the clinical translation of eco-AgNPs for cancer therapy (Created by the author and adapted from [57, 79–81]).

Despite the remarkable preclinical promise and versatility of eco-AgNPs in cancer therapy, their widespread clinical translation faces substantial and multifaceted challenges. There is a clear paradox between the high efficacy observed in laboratory settings and the numerous practical, engineering, and regulatory hurdles that impede their journey from bench to bedside. The complex pathway from laboratory discovery to clinical implementation, along with corresponding challenges and proposed solutions, is illustrated in Figure 2. Overcoming these barriers is as crucial as continuing to discover new therapeutic effects. A primary concern revolves around the potential for off-target toxicity and the long-term accumulation of AgNPs in vital organs, such as the liver. While silver is not traditionally considered a cumulative poison, prolonged exposure can lead to undesired effects, necessitating rigorous and comprehensive toxicological studies, particularly *in vivo* assessments, to fully understand their safety profiles and ensure biocompatibility for human use [82]. Additionally, achieving large-scale, cost-effective, and consistent production of eco-AgNPs with uniform physicochemical properties remains a significant challenge for clinical application. Scalability remains a particular hurdle for eco-AgNPs, as microbial- or plant-based synthesis lacks the rapid, high-yield production of chemical methods [83]. However, advances in bioreactor optimization and standardized extraction protocols are narrowing this gap. The long-term stability of nanoparticles during storage, including issues like aggregation or oxidation, is often neglected but critical for maintaining their therapeutic efficacy [79].

The regulatory landscape for nanomedicines is complex and stringent, requiring extensive data on safety, efficacy, and manufacturing quality. Specific issues like endotoxin contamination, which can lead to early failure in clinical trials, also pose hurdles, as current quantification assays are often inadequate [57, 79]. Furthermore, a complete understanding of the precise intracellular, paracellular, and transcellular pathways of nanodrugs across biological membranes is still lacking, which can hinder efficient cellular internalization into tumor cells. Achieving optimal and sustained, targeted drug release *in vivo* without premature leakage remains a significant problem. The dense tumor stroma, or desmoplasia, particularly prevalent in cancers like pancreatic adenocarcinoma, presents a formidable physical barrier that impedes drug penetration and distribution within the tumor [57, 65].

Cancer’s inherent genetic and functional heterogeneity within single tumors, coupled with cancer cells’ remarkable ability to develop resistance mechanisms, poses a continuous challenge even to novel AgNP-based therapies [75, 80]. Additionally, predicting individual patient responses to nanomedicines remains difficult, and there is potential for unexpected allergic or adverse reactions in diverse patient populations, which complicates broad clinical application [57]. The complexity of these challenges necessitates a broad spectrum of specialized knowledge spanning chemistry, materials science, biology, engineering, medicine, and regulatory science. This underscores the critical need for robust, multi-disciplinary, and collaborative research ecosystems to accelerate the safe and effective clinical translation of eco-AgNPs.

To bridge the gap between bench and bedside, recent international efforts have focused on harmonizing standards for nanomedicines. The OECD’s 2025 report on the safety testing of manufactured nanomaterials [84] and the new guidance on assessing accumulation potential [85] provide critical frameworks for addressing dosing regimens and toxicokinetics. These guidelines aim to harmonize safety assessments across sectors and are particularly relevant for mitigating the batch-to-batch variability often associated with green synthesis. Concurrently, ISO/TS 20660:2019 establishes rigorous benchmarks for characterizing AgNPs, detailing measurement methods for essential attributes such as primary particle size, zeta potential, and total silver content [86]. Adhering to these specifications is vital for ensuring the reproducibility of eco-AgNPs in oncological applications.

From a regulatory perspective, the FDA’s guidance on drug products containing nanomaterials emphasizes a risk-based approach to characterization, focusing on critical quality attributes (CQAs) and the potential for altered biodistribution and long-term toxicity [87]. Similarly, the EMA’s *Regulatory Science Strategy to 2025* [88] and *Horizon Scanning Report* [89] highlight the necessity of quality-by-design principles and the development of specific evaluation pathways for complex nanomedicines, including stimuli-responsive systems. These evolving regulatory frameworks outline a clearer roadmap for verifying the safety and quality of eco-AgNPs, thereby facilitating their transition from laboratory discovery to clinical trials.

Looking toward the future, continued efforts are imperative to develop safer, more efficient, and scalable synthesis methods, with a strong emphasis on green synthesis approaches to enhance biocompatibility and reduce environmental impact. Future research will focus on designing novel functionalization strategies that achieve even greater specificity, further reduce off-target toxicity, and enable sophisticated stimuli-responsive drug release, allowing for precise control over drug delivery at the tumor site [81]. Moving beyond simple synthesis, a novel Frontier lies in 'corona engineering'-manipulating the biological feedstocks during green synthesis to selectively incorporate specific plant proteins or metabolites into the nanoparticle coating. This would effectively encode targeting or stimuli-responsive properties directly into the eco-AgNP’s native surface during synthesis, reducing the need for complex post-synthesis chemical modifications. There is immense potential in exploring synergistic effects by combining eco-AgNPs with conventional treatments such as chemotherapy and radiotherapy, as well as emerging therapies including immunotherapy and gene therapy. AgNPs have been shown to intensify the effect of chemotherapeutic agents, and combination therapies targeting cancer stem cell genes represent a sophisticated avenue for overcoming drug resistance and enhancing overall therapeutic efficacy, particularly in challenging cancers like non-small cell lung cancer [90]. The continued development of eco-AgNP-based theranostic agents for real-time, non-invasive monitoring of drug delivery, biodistribution, and treatment response is crucial. Furthermore, expanding the use of high-throughput 'omics’ technologies, including proteogenomics and metabolomics, is essential to unravel the complex molecular and metabolic changes induced by eco-AgNPs in cancer cells, including those currently unknown (as indicated by the question mark in Figure 1). This deeper understanding is crucial for developing more robust, effective, and adaptive anticancer therapies that can circumvent resistance mechanisms [80].

Future strategies must focus on developing innovative approaches to actively modulate or overcome the physical and biological barriers posed by the tumor microenvironment, such as disrupting desmoplasia, to improve AgNP penetration and therapeutic efficacy [80]. The future of eco-AgNPs in oncology will involve their further integration into personalized medicine frameworks by leveraging advanced technologies to overcome key translational hurdles. For instance, AI-driven diagnostics can analyze complex patient data to predict treatment outcomes, directly addressing the challenges of tumor heterogeneity and unpredictable patient responses. Concurrently, “organ-on-a-chip” platforms using patient-derived cells can provide more accurate preclinical screening for both efficacy and toxicity, helping to resolve biocompatibility concerns before human trials. This signifies an evolution from viewing eco-AgNPs merely as a “drug” to conceptualizing them as sophisticated, multi-functional systems that can intelligently interact with the complex biological environment. The success of eco-AgNPs in oncology hinges not just on the silver itself, but on the entire engineered nanoplatform surrounding it, emphasizing design over mere discovery [12, 81, 91].

## Discussion

Silver nanoparticles represent a transformative therapeutic platform in precision oncology, offering multi-modal anticancer mechanisms including reactive oxygen species generation, apoptosis induction, and cell cycle arrest. Eco-AgNPs present compelling advantages over chemically synthesized variants through ecofriendly synthesis, reduced systemic toxicity, and inherent biomolecular coronas that facilitate functionalization. Advanced targeting strategies enable selective tumor accumulation, while integration with biomarker-guided applications facilitates personalized treatment by exploiting cancer-specific molecular signatures. AgNP-based theranostics advance the field by combining diagnostic imaging with therapeutic delivery, enabling real-time treatment monitoring and adaptive therapy optimization. However, substantial challenges remain, including long-term toxicity concerns, clinical translation hurdles involving scalability and regulatory compliance, and tumor heterogeneity with associated physical barriers.

Future research priorities include optimizing green synthesis methods, developing novel functionalization strategies, and exploring combination therapies. Advanced omics technologies will provide crucial mechanistic insights for designing robust treatments. Successful clinical translation requires viewing eco-AgNPs as versatile engineered platforms demanding multidisciplinary collaboration. With continued innovation, eco-AgNPs hold tremendous potential to revolutionize cancer treatment and improve patient outcomes worldwide.

## References

[1] Jamalinia M Weiskirchen R . Advances in personalized medicine: translating genomic insights into targeted therapies for cancer treatment. Ann Transl Med (2025) 13:18. 10.21037/atm-25-34 40438512 PMC12106117

[2] Takáč P Michalková R Čižmáriková M Bedlovičová Z Balážová Ľ Takáčová G . The role of silver nanoparticles in the diagnosis and treatment of cancer: are there any perspectives for the future? Life (2023) 13:13. 10.3390/life13020466 36836823 PMC9965924

[3] Hamzah HM Salah RF Maroof MN . *Fusarium mangiferae* as new cell factories for producing silver nanoparticles. J Microbiol Biotechnol (2018) 28:1654–63. 10.4014/jmb.1806.06023 30196593

[4] Fattah B Arif H Hamzah H . Antimicrobial and antibiofilm activity of biosynthesized silver nanoparticles against beta-lactamase-resistant *Enterococcus faecalis* . Appl Biochem Biotechnol (2022) 194:2036–46. 10.1007/s12010-022-03805-y 35015218

[5] Mohammed L Hamzah H . *Streptomyces pratensis*-mediated fabrication of silver nanoparticles and its applications as antimicrobial and anticancer. Bionanoscience (2024) 14:1021–32. 10.1007/s12668-024-01334-y

[6] Bakhtyar R Tofiq R Hamzah H Qurbani K . *Fabricated Fusarium* species-mediated nanoparticles against Gram-negative pathogen. World Acad Sci J (2025) 7. (Review). 10.3892/wasj.2024.289

[7] Abramenko N Semenova M Khina A Zherebin P Krutyakov Y Krysanov E The toxicity of coated silver nanoparticles and their stabilizers towards *Paracentrotus lividus* sea urchin embryos. Nanomaterials (2022) 12:12. 10.3390/nano12224003 36432289 PMC9695290

[8] Sedlakova-Kadukova J Sincak M Demčakova V . Does the silver nanoparticles production route affect the proliferation of antibiotic resistance in soil ecosystem? Antibiotics (2025) 14:14. 10.3390/antibiotics14010015 39858301 PMC11762139

[9] Ahmed A-A Hamzah H Maaroof M . Analyzing formation of silver nanoparticles from the filamentous fungus *Fusarium oxysporum* and their antimicrobial activity. Turk J Biol (2018) 42:54–62. 10.3906/biy-1710-2 30814870 PMC6353250

[10] Hussein S Sulaiman S Ali S Pirot R Qurbani K Hamzah H Synthesis of silver nanoparticles from *Aeromonas caviae* for antibacterial activity and *in vivo* effects in rats. Biol Trace Elem Res (2023) 202:2764–75. 10.1007/s12011-023-03876-w 37752375

[11] Durán N Fávaro WJ Alborés S Costa TS Tasic L . Biogenic silver nanoparticles capped with proteins: timed knowledge and perspectives. J Braz Chem Soc (2023) 34:897–905. 10.21577/0103-5053.20230062

[12] Fernandes DA . Review on metal-based theranostic nanoparticles for cancer therapy and imaging. Technol Cancer Res Treat (2023) 22:22. 10.1177/15330338231191493 37642945 PMC10467409

[13] Mousavi SM Hashemi SA Ghasemi Y Atapour A Amani AM Savar Dashtaki A Green synthesis of silver nanoparticles toward bio and medical applications: review study. Artif Cells Nanomed Biotechnol (2018) 46:S855–72. 10.1080/21691401.2018.1517769 30328732

[14] Qurbani K Hussein S Hamzah H Sulaiman S Pirot R Motevaseli E Synthesis of silver nanoparticles by *Raoultella planticola* and their potential antibacterial activity against multidrug-resistant isolates. Iran J Biotechnol (2022) 20:75–83. 10.30498/ijb.2022.298773.3121 38344316 PMC10858361

[15] Gutiérrez Coronado O Sandoval Salazar C Muñoz Carrillo JL Gutiérrez Villalobos OA Miranda Beltrán MD Soriano Hernández AD Functionalized nanomaterials in cancer treatment: a review. Int J Mol Sci (2025) 26:26. 10.3390/ijms26062633 40141274 PMC11942109

[16] Shannahan J . The biocorona: a challenge for the biomedical application of nanoparticles. Nanotechnol Rev (2017) 6:345–53. 10.1515/ntrev-2016-0098 29607287 PMC5875931

[17] Lim HK Asharani PV Hande MP . Enhanced genotoxicity of silver nanoparticles in DNA repair deficient mammalian cells. Front Genet (2012) 3:3. 10.3389/fgene.2012.00104 22707954 PMC3374476

[18] AlSawaftah NM Awad NS Pitt WG Husseini GA . pH-responsive nanocarriers in cancer therapy. *Polymers* (Basel) (2022) 14:14. 10.3390/polym14050936 35267759 PMC8912405

[19] Buttacavoli M Albanese NN Di Cara G Alduina R Faleri C Gallo M Anticancer activity of biogenerated silver nanoparticles: an integrated proteomic investigation. Oncotarget (2017) 9(11):9685–705. 10.18632/oncotarget.23859 29515763 PMC5839394

[20] Ullah I Khalil AT Ali M Iqbal J Ali W Alarifi S Green-synthesized silver nanoparticles induced apoptotic cell death in MCF-7 breast cancer cells by generating reactive oxygen species and activating caspase 3 and 9 enzyme activities. Oxid Med Cell Longev (2020) 2020:2020. 10.1155/2020/1215395 33082906 PMC7559220

[21] Khan MS Alomari A Tabrez S Hassan I Wahab R Bhat SA Anticancer potential of biogenic silver nanoparticles: a mechanistic study. Pharmaceutics (2021) 13:13. 10.3390/pharmaceutics13050707 34066092 PMC8151171

[22] Danışman-Kalındemirtaş F Kariper İA Üstündağ H Özsoy C Erdem-Kuruca S . Antiproliferative Effects of 5FU-AgNPs On Different Breast Cancer Cells. J Taibah Univ Sci (2024). p. 18. 10.1080/16583655.2024.2354573

[23] An X Yu W Liu J Tang D Yang L Chen X . Oxidative cell death in cancer: mechanisms and therapeutic opportunities. Cell Death Dis (2024) 15:15. 10.1038/s41419-024-06939-5 39090114 PMC11294602

[24] Kabir SR Islam F Asaduzzaman AKM . Biogenic silver/silver chloride nanoparticles inhibit human cancer cells proliferation *in vitro* and Ehrlich ascites carcinoma cells growth *in vivo* . Sci Rep (2022) 12:12. 10.1038/s41598-022-12974-z 35618812 PMC9135710

[25] Vahabirad M Daei S Abbasalipourkabir R Ziamajidi N . Anticancer action of silver nanoparticles in SKBR3 breast cancer cells through promotion of oxidative stress and apoptosis. Biomed Res Int (2024) 2024:7145339. 10.1155/2024/7145339 38410788 PMC10896653

[26] Veeragoni D Deshpande S Rachamalla HK Ande A Misra S Mutheneni SR . *In vitro* and *in vivo* anticancer and genotoxicity profiles of green synthesized and chemically synthesized silver nanoparticles. ACS Appl Bio Mater (2022) 5:2324–39. 10.1021/acsabm.2c00149 35426672

[27] Yasin D Sami N Afzal B Zaki A Naaz H Husain S Biogenic nanoparticles: understanding their potential role in cancer theranostics. Next Nanotechnol (2025) 8:8. 10.1016/j.nxnano.2025.100149

[28] Ratan ZA Haidere MF Nurunnabi MD Shahriar SM Ahammad AS Shim YY Green chemistry synthesis of silver nanoparticles and their potential anticancer effects. *Cancers* (Basel) (2020) 12:12. 10.3390/cancers12040855 32244822 PMC7226404

[29] Ahire JH Wang Q Tao Y Chao Y Bao Y. Amine-terminated silver nanoparticles exhibit potential for selective targeting of triple-negative breast cancer. Appl Nano (2024) 5:227–44. 10.3390/applnano5040015

[30] Spagnoletti FN Kronberg F Spedalieri C Munarriz E Giacometti R . Protein corona on biogenic silver nanoparticles provides higher stability and protects cells from toxicity in comparison to chemical nanoparticles. J Environ Manage (2021) 297:297. 10.1016/j.jenvman.2021.113434 34400389

[31] Subhan MA Yalamarty SS Filipczak N Parveen F Torchilin VP . Recent advances in tumor targeting *via* epr effect for cancer treatment. J Pers Med (2021) 11:11. 10.3390/jpm11060571 34207137 PMC8234032

[32] Chen Z Kankala RK Long L Xie S Chen A Zou L . Current understanding of passive and active targeting nanomedicines to enhance tumor accumulation. Coord Chem Rev (2023) 481:215051. 10.1016/j.ccr.2023.215051

[33] Shinde VR Revi N Murugappan S Singh SP Rengan AK . Enhanced permeability and retention effect: a key facilitator for solid tumor targeting by nanoparticles. Photodiagnosis Photodyn Ther (2022) 39:102915. 10.1016/j.pdpdt.2022.102915 35597441

[34] Vagena IA Malapani C Gatou MA Lagopati N Pavlatou EA . Enhancement of EPR effect for passive tumor targeting: current status and future perspectives. *Appl Sci* (Basel) (2025) 15:15. 10.3390/app15063189

[35] Chen Z Kankala RK Yang Z Li W Xie S Li H Antibody-based drug delivery systems for cancer therapy: mechanisms, challenges, and prospects. Theranostics (2022) 12:3719–46. 10.7150/thno.72594 35664074 PMC9131265

[36] Sitia L Sevieri M Signati L Bonizzi A Chesi A Mainini F HER-2-targeted nanoparticles for breast cancer diagnosis and treatment. *Cancers* (Basel) (2022) 14:14. 10.3390/cancers14102424 35626028 PMC9139811

[37] Crintea A Constantin AM Motofelea AC Crivii CB Velescu MA Coşeriu RL Targeted EGFR nanotherapy in non-small cell lung cancer. J Funct Biomater (2023) 14:14. 10.3390/jfb14090466 37754880 PMC10532491

[38] Hussein S Qurbani K Hamzah H Ali S Ahmed SK . Biotechnology breakthroughs: revolutionizing oral cancer treatment. Oral Oncol Rep (2024) 10:10. 10.1016/j.oor.2024.100404

[39] Jiang S Wang X Zhang Z Sun L Pu Y Yao H CD20 monoclonal antibody targeted nanoscale drug delivery system for doxorubicin chemotherapy: an *in vitro* study of cell lysis of CD20-positive Raji cells. Int J Nanomedicine (2016) 11:5505–18. 10.2147/IJN.S115428 27843311 PMC5098746

[40] Chiu ML Goulet DR Teplyakov A Gilliland GL . Antibody structure and function: the basis for engineering therapeutics. Antibodies (2019) 8:8. 10.3390/antib8040055 31816964 PMC6963682

[41] Iroegbu AOC Teffo ML Sadiku ER . Cancer therapy with engineered nanozymes: from molecular design to tumour-responsive catalysis. Nanomedicine (2025) 20:1799–817. 10.1080/17435889.2025.2520736 40531138 PMC12239811

[42] Chen W Jarzyna PA Van Tilborg GA Nguyen VA Cormode DP Klink A RGD peptide functionalized and reconstituted high‐density lipoprotein nanoparticles as a versatile and multimodal tumor targeting molecular imaging probe. FASEB J (2010) 24:1689–99. 10.1096/fj.09-139865 20075195 PMC2874482

[43] Hamilton AM Aidoudi-Ahmed S Sharma S Kotamraju VR Foster PJ Sugahara KN Nanoparticles coated with the tumor-penetrating peptide iRGD reduce experimental breast cancer metastasis in the brain. J Mol Med (2015) 93:991–1001. 10.1007/s00109-015-1279-x 25869026 PMC4807972

[44] Lorenzoni S Rodríguez-Nogales C Blanco-Prieto MJ . Targeting tumor microenvironment with RGD-functionalized nanoparticles for precision cancer therapy. Cancer Lett (2025) 614. 10.1016/j.canlet.2025.217536 39924081

[45] Kang S Lee S Park S . iRGD peptide as a tumor-penetrating enhancer for tumor-targeted drug delivery. *Polymers* (Basel) (2020) 12:12. 10.3390/POLYM12091906 32847045 PMC7563641

[46] Zhou J Rossi J . Aptamers as targeted therapeutics: current potential and challenges. Nat Rev Drug Discov (2017) 16:181–202. 10.1038/nrd.2016.199 27807347 PMC5700751

[47] Odeh F Nsairat H Alshaer W Ismail MA Esawi E Qaqish B Aptamers chemistry: chemical modifications and conjugation strategies. Molecules (2020) 25:25. 10.3390/molecules25010003 31861277 PMC6982925

[48] Hermanson GT . Bioconjugate Techniques. Academic Press (2013). ISBN 978-0-12-382239-0. 10.1016/C2009-0-64240-9

[49] Walkey CD Chan WCW . Understanding and controlling the interaction of nanomaterials with proteins in a physiological environment. Chem Soc Rev (2012) 41:2780–99. 10.1039/c1cs15233e 22086677

[50] Shi L Zhang J Zhao M Tang S Cheng X Zhang W Effects of polyethylene glycol on the surface of nanoparticles for targeted drug delivery. Nanoscale (2021) 13:10748–64. 10.1039/D1NR02065J 34132312

[51] Alaarg A Senders ML Varela-Moreira A Pérez-Medina C Zhao Y Tang J A systematic comparison of clinically viable nanomedicines targeting HMG-CoA reductase in inflammatory atherosclerosis. J Control Release (2017) 262:47–57. 10.1016/j.jconrel.2017.07.013 28700897

[52] Chu S Shi X Tian Y Gao F . pH-responsive polymer nanomaterials for tumor therapy. Front Oncol (2022) 12:12. 10.3389/fonc.2022.855019 35392227 PMC8980858

[53] Liu Y Si L Jiang Y Jiang S Zhang X Li S Design of pH-responsive nanomaterials based on the tumor microenvironment. Int J Nanomedicine (2025) 20:705–21. 10.2147/IJN.S504629 39845771 PMC11752822

[54] Gao W Chan JM Farokhzad OC . PH-responsive nanoparticles for drug delivery. Mol Pharm (2010) 7:1913–20. 10.1021/mp100253e 20836539 PMC3379544

[55] Liu D Jin C Shan F He J Wang F . Synthesizing BaTiO_3_ nanostructures to explore morphological influence, kinetics, and mechanism of piezocatalytic dye degradation. ACS Appl Mater Inter (2020) 12:17443–51. 10.1021/acsami.9b23351 32195558

[56] Mi Y Wolfram J Mu C Liu X Blanco E Shen H Enzyme-responsive multistage vector for drug delivery to tumor tissue. Pharmacol Res (2016) 113:92–9. 10.1016/j.phrs.2016.08.024 27546164 PMC5107143

[57] Mundekkad D Cho WC . Nanoparticles in clinical translation for cancer therapy. Int J Mol Sci (2022) 23:23. 10.3390/ijms23031685 35163607 PMC8835852

[58] Tong F Wang Y Gao H . Progress and challenges in the translation of cancer nanomedicines. Curr Opin Biotechnol (2024) 85:103045. 10.1016/j.copbio.2023.103045 38096768

[59] Laib I Gheraissa N Benaissa A Benkhira L Azzi M Benaissa Y Tailoring innovative silver nanoparticles for modern medicine: the importance of size and shape control and functional modifications. Mater Today Bio (2025) 33:33. 10.1016/j.mtbio.2025.102071 40727080 PMC12302930

[60] Rituraj PRS Wahlang J Pal Y Chaitanya MV Saxena S . Precision oncology: transforming cancer care through personalized medicine. Med Oncol (2025) 42:246. 10.1007/s12032-025-02817-y 40488843

[61] Lan H Jamil M Ke G Dong N . The role of nanoparticles and nanomaterials in cancer diagnosis and treatment: a comprehensive review. Am J Cancer Res (2023) 13(12):5751–84. 38187049 PMC10767363

[62] Wang B Hu S Teng Y Chen J Wang H Xu Y Current advance of nanotechnology in diagnosis and treatment for malignant tumors. Signal Transduct Target Ther (2024) 9:200. 10.1038/s41392-024-01889-y 39128942 PMC11323968

[63] Skóra B Piechowiak T Szychowski KA . Epidermal growth factor-labeled liposomes as a way to target the toxicity of silver nanoparticles into EGFR-overexpressing cancer cells *in vitro* . Toxicol Appl Pharmacol (2022) 443:116009. 10.1016/j.taap.2022.116009 35385781

[64] Kumar G Nandakumar K Mutalik S Rao CM . Biologicals to direct nanotherapeutics towards HER2-positive breast cancers. Nanomedicine (2020) 27:102197. 10.1016/j.nano.2020.102197 32275958

[65] Zhu L Staley C Kooby D El-Rays B Mao H Yang L . Current status of biomarker and targeted nanoparticle development: the precision oncology approach for pancreatic cancer therapy. Cancer Lett (2017) 388:139–48. 10.1016/j.canlet.2016.11.030 27916607 PMC5318282

[66] Villalobos Gutiérrez PT Muñoz Carrillo JL Sandoval Salazar C Viveros Paredes JM Gutiérrez Coronado O . Functionalized metal nanoparticles in cancer therapy. Pharmaceutics (2023) 15:15. 10.3390/pharmaceutics15071932 37514119 PMC10383728

[67] Morais M Machado V Dias F Figueiredo P Palmeira C Martins G Glucose-functionalized silver nanoparticles as a potential new therapy agent targeting hormone-resistant prostate cancer cells. Int J Nanomedicine (2022) 17:4321–37. 10.2147/IJN.S364862 36147546 PMC9489222

[68] Liang J Zeng F Zhang M Pan Z Chen Y Zeng Y Green synthesis of hyaluronic acid-based silver nanoparticles and their enhanced delivery to CD44+ cancer cells. RSC Adv (2015) 5:43733–40. 10.1039/c5ra03083h

[69] Metrangolo V Blomquist MH Dutta A Gårdsvoll H Krigslund O Nørregaard KS Targeting uPAR with an antibody-drug conjugate suppresses tumor growth and reshapes the immune landscape in pancreatic cancer models. Sci Adv (2025) 11(3):eadq0513. 10.1126/sciadv.adq0513 39823326 PMC11740940

[70] O’Neill RS Stoita A . Biomarkers in the diagnosis of pancreatic cancer: are we closer to finding the golden ticket? World J Gastroenterol (2021) 27:4045–87. 10.3748/wjg.v27.i26.4045 34326612 PMC8311531

[71] Neamatallah MA Fatouh AA Eldeib D Nematallah A Ammar O El-Badrawy MK Effect of chemotherapy combination with AgNPs, AgNO_3_, and NaHCO_3_ on the progression of non-small lung cancer *via* targeting cancer stem cell genes (2025). 10.21203/rs.3.rs-6033175/v1

[72] Kumbhakar DV Thakkar L Akhand C Sharaf S Vemuganti GK . Nanomaterials targeting cancer stem cells to overcome drug resistance and tumor recurrence. Front Oncol (2025) 15:15. 10.3389/fonc.2025.1499283 40548119 PMC12178904

[73] Passaro A Al Bakir M Hamilton EG Diehn M André F Roy-Chowdhuri S Cancer biomarkers: emerging trends and clinical implications for personalized treatment. Cell (2024) 187:1617–35. 10.1016/j.cell.2024.02.041 38552610 PMC7616034

[74] Kravets V Almemar Z Jiang K Culhane K Machado R Hagen G Imaging of biological cells using luminescent silver nanoparticles. Nanoscale Res Lett (2016) 11:11. 10.1186/s11671-016-1243-x 26781288 PMC4717127

[75] Raja G Jang YK Suh JS Kim HS Ahn SH Kim TJ . Microcellular environmental regulation of silver nanoparticles in cancer therapy: a critical review. *Cancers* (Basel) (2020) 12:1–33. 10.3390/cancers12030664 32178476 PMC7140117

[76] AlDoughaim M AlSuhebany N AlZahrani M AlQahtani T AlGhamdi S Badreldin H Cancer biomarkers and precision oncology: a review of recent trends and innovations. Clin Med Insights Oncol (2024) 18:18. 10.1177/11795549241298541 39559827 PMC11571259

[77] Lin Z Wang L Xing Z Wang F Cheng X . Update on combination strategies of PARP inhibitors. Cancer Control (2024) 31:31. 10.1177/10732748241298329 39500600 PMC11539152

[78] Gao F Liu F Wang J Bi J Zhai L Li D . Molecular probes targeting HER2 PET/CT and their application in advanced breast cancer. J Cancer Res Clin Oncol (2024) 150:150. 10.1007/s00432-023-05519-y 38466436 PMC10927773

[79] El-Tanani M Satyam SM Rabbani SA El-Tanani Y Aljabali AA Al Faouri I Revolutionizing drug delivery: the impact of advanced materials science and technology on precision medicine. Pharmaceutics (2025) 17:17. 10.3390/pharmaceutics17030375 40143038 PMC11944361

[80] Raies S Rehman U Siddiqua A Wahab S Gupta G Goh KW Silver nanoparticles: forging a new frontline in lung cancer therapy. Biomater Adv (2025) 177:214395. 10.1016/j.bioadv.2025.214395 40577936

[81] Ranjbar S Bakhtiari A Khosravi N Ashkavandi SJ Azamian F Alijaniha M Silver Nanoparticles: Biomedical Applications and Future Perspectives. Tbilisi, Georgia: The University of Georgia Publishing House. (2024). p. 6. 10.61186/jcc.6.3.2

[82] Sati A Ranade TN Mali SN Ahmad Yasin HK Pratap A . Silver nanoparticles (AgNPs): comprehensive insights into bio/synthesis, key influencing factors, multifaceted applications, and toxicity-A 2024 update. ACS Omega (2025) 10:7549–82. 10.1021/acsomega.4c11045 40060826 PMC11886731

[83] Fahim M Shahzaib A Nishat N Jahan A Bhat TA Inam A . Green synthesis of silver nanoparticles: a comprehensive review of methods, influencing factors, and applications. JCIS Open (2024) 16:16. 10.1016/j.jciso.2024.100125

[84] OECD. Report on the implementation of the OECD recommendation on the safety testing and assessment of manufactured nanomaterials. council document C(2025)72. Paris: Organisation for Economic Co-operation and Development (2025).

[85] OECD. Guidance document on assessing the apparent accumulation potential of nanomaterials. In: Series on Testing and Assessment No. 406, ENV/CBC/MONO(2025)3. Paris: Organisation for Economic Co-operation and Development (2025).

[86] ISO. ISO/TS 20660:2019 Nanotechnologies - Antibacterial Silver Nanoparticles - Specification of Characteristics and Measurement Methods. Geneva: International Organization for Standardization (2019).

[87] FDA. Drug Products, Including Biological Products, that Contain Nanomaterials: Guidance for Industry. Silver spring, MD: U.S. Food and Drug Administration, Center for Drug Evaluation and Research CDER and Center for Biologics Evaluation and Research CBER (2022).

[88] European medicines agency. EMA Regulatory Science to 2025: Strategic Reflection. Amsterdam: European Medicines Agency (2020). EMA/110706/2020.

[89] European medicines agency and heads of medicines agencies. Nanotechnology-Based Medicinal Products for Human Use: EU-IN Horizon Scanning Report. Amsterdam: European Medicines Agency (2025). EMA/20989/2025/Rev. 1.

[90] Darwish A Sándor N Szenti I Marosvölgyi T Juhász K Rónavári A Highly stable antitumor silver-lipid nanoparticles optimized for targeted therapy. Int J Nanomedicine (2025) 20:1351–66. 10.2147/IJN.S498208 39911260 PMC11796454

[91] Choudhary F Naikoo UM Rizwan A Kaur J Abdin MZ Farooqi H Innovation in lung cancer management from herbal nanomedicine to artificial intelligence, J. Nanotheranostics (2025). 6:19. 10.3390/jnt6030019

